# Sensing with Nanopores and Aptamers: A Way Forward

**DOI:** 10.3390/s20164495

**Published:** 2020-08-11

**Authors:** Lucile Reynaud, Aurélie Bouchet-Spinelli, Camille Raillon, Arnaud Buhot

**Affiliations:** Univ. Grenoble Alpes, CEA, CNRS, IRIG, SyMMES, F-38000 Grenoble, France; lucile.reynaud@cea.fr (L.R.); aurelie.bouchet-spinelli@cea.fr (A.B.-S.); camille.raillon@cea.fr (C.R.)

**Keywords:** nanopores, nanopipettes, nanochannels, aptamers, biological pores, translocation, single-molecule, biomimetic nanopores

## Abstract

In the 90s, the development of a novel single molecule technique based on nanopore sensing emerged. Preliminary improvements were based on the molecular or biological engineering of protein nanopores along with the use of nanotechnologies developed in the context of microelectronics. Since the last decade, the convergence between those two worlds has allowed for biomimetic approaches. In this respect, the combination of nanopores with aptamers, single-stranded oligonucleotides specifically selected towards molecular or cellular targets from an in vitro method, gained a lot of interest with potential applications for the single molecule detection and recognition in various domains like health, environment or security. The recent developments performed by combining nanopores and aptamers are highlighted in this review and some perspectives are drawn.

## 1. Introduction

Nanopore sensing is based on the Coulter counter [1] principle proposed in 1953, a resistive sensing device able to count and size objects going through an aperture. Nanopore technology comes from the merging of the Coulter counter and single-channel electrophysiology [2], which is the study of transmembrane current through lipid bilayers. In the 90s, thanks to the concomitant development of biotechnologies and nanotechnologies, studies about single molecule translocation through a single biological pore in a planar lipid bilayer emerged [3]. In the beginning of the 2000s, the first solid-state nanopores for studies of single-molecule translocation were fabricated [4].

**Figure 1:**
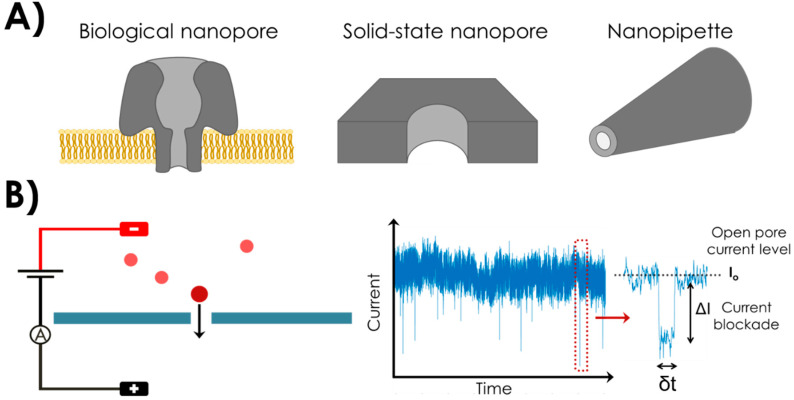
(**A**) Illustration of a biological nanopore in a lipid bilayer (left) a solid-state nanopore fabricated in an insulating membrane (middle) and a nanopipette (right). (**B**) Solid-state nanopore principle. A dielectric membrane separates two electrolytes reservoirs connected solely through the nanopore. Electrodes are placed at each side of the membrane and a voltage is applied. Measured ionic current results from the electrolyte charges moving through the nanopore.

A typical nanopore device consists in a nanometric aperture in a dielectric membrane between two reservoirs of a conductive electrolyte solution. An illustration of a biological and a solid-state nanopore and its principle is given in Figure 1. The nanopore is the only contact between the two reservoirs. Electrodes are immersed on each side of the membrane, and an electric current flowing through the nanopore is established when a voltage is applied across those electrodes [5]. This steady-state current is called open-pore current. Typically, the current is monitored for different applied voltages and a linear current-voltage (I-V) curve is obtained. The nanopore conductance and size can be calculated from this curve [5]. 

The principle of single molecule detection is depicted in Figure 1B. A voltage is applied between the two electrodes. When a biomolecule, such as DNA or protein, is added into one of the electrolyte reservoirs, it is electrophoretically driven through the nanopore (translocation) and a disruption in the current signal is observed. Consecutive disruptions correspond to successive biomolecule translocations in the nanopore. The current blockage amplitude ΔI is the difference between the open pore current and the current level when the biomolecule is inside the nanopore. The dwell time δt corresponds to the duration of the translocation event. A statistical analysis of the current blockades amplitudes (ΔI) and durations (δt) can provide information on the biomolecule such as its volume, charge or conformation [5,6,7,8,9]. A statistical analysis of time distribution between two consecutive translocation is informative on the concentration of the analyte [10].

More recently, novel configuration of sensing nanopores emerged based on nanopipettes [11] (see Figure 1A). Those are made of glass with one electrode inside and the other in the surrounding medium. Such configuration is potentially easier to fabricate than traditional nanopores in flat membranes.

Even if the sensing of single molecules is the most relevant and frequent use of nanopores, based on their functionalization novel applications are emerging like ionic gates, robots, logic functions. Those potential applications extend the interest of the research domains of functional nanopores.

Several reviews have focused on nanopores and their single molecule detection principle and applications [5,11,12,13,14,15,16,17,18,19,20,21,22,23,24,25,26,27]. As mentioned previously, in most cases, the use of functional molecules brings new capabilities in term of detection, recognition, selectivity and even function to the nanopores. In this respect, aptamers [28,29], small oligonucleotide sequences, specifically selected towards a target like small ions, biomarkers, proteins or even cells are good candidates. Their small size is compatible with nano-confinement and their ease of chemical modification confers straightforward functionalization on surfaces. Their strong affinity towards a large kind of targets allows for versatility in the applications. The 3D conformation required for the target recognition may also be used as a trigger for conformational changes and further activation of the nanopores. Finally, their stability and cost of production are also compatible with a potential future industrialization. Based on all those ingredients, combining nanopores with aptamers has been recognized to present only advantages. In this review, we aimed at presenting the recent literature on this domain. First, we will describe the different configurations of nanopores encountered from biological to synthetic and more recent biomimetic pores and from pores in flat membranes or nanopipettes. Then, we will describe the different use of aptamers in combination with nanopores: for fundamental researches on their conformation and mode of action, for single molecule detection from their affinity and finally first approaches and perspectives for the use of their conformational switch in order to bring functionality to the pores.

## 2. Nanopores and Nanopipettes

### 2.1. Biological Nanopores

**Figure 2:**
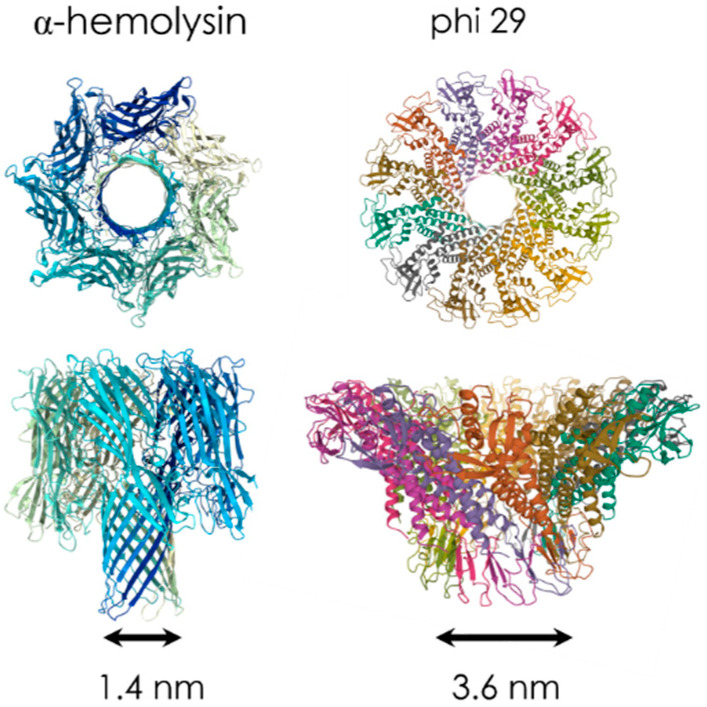
α-hemolysin (α-HL) nanopore structure (PDB: 7AHL) and phi29 motor nanopore structure (PDB: 1JNB).

Biological pores are found in Nature as proteins that act as transport channels through the membrane of cells. They take various forms and purposes [30], such as ion channel proteins (for ionic transport), porins and aquaporins (for water-soluble components and water), nuclear pore complexes [31] (transport of oligonucleotides and proteins), pore-forming toxin peptides (that can trigger the lysis of the cell) or viral pores (for the transport of viral DNA into the infected cell). Those proteins or protein assemblies are inserted in a lipid bilayer. One of the most popular biological nanopores among single-molecule researchers is α-hemolysin (α-HL). It was the biological nanopore used in 1996 by Kasianowicz et al. for the first demonstration of RNA and DNA single-molecule detection with a nanopore [3]. α-HL is a bacterial pore secreted from *Staphylococcus aureus* with a well-known crystal structure [32], the protein has a 10 × 10 nm^2^ cylindrical shape with a lumen ranging from 4.6 nm to 1.4 nm in diameter. This pore enables highly reproducible DNA translocation experiments. Some other biological nanopores [33] commonly used for translocation of nucleic acids, small peptides or unfolded proteins are Outer membrane protein G OmpG [34] (with an internal diameter of 1.3 nm), *Mycobacterium smegmatis* porin A MspA [35] (1.2 nm) and Aerolysin AeL [36,37] (1.0 nm). However, for sensing larger molecules such as proteins, different biological nanopores with a wider diameter are also used, such as Cytolysin A ClyA [38,39] (diameter 3.3 nm) and phi29 motor pores [40,41] (3.6 nm). Examples of biological nanopores with their dimension are illustrated on Figure 2.

Biological nanopores have the advantages to offer highly reproducible translocation results thanks to a well-defined pore structure. The lipid bilayer in which they are embedded offers low electrical noise [23,25]. Their chemical structure can be tuned with the addition of functional groups thanks to genetic or molecular engineering [19] and the variety of available biological pores increases over time [42]. However, they present a low mechanical and chemical stability over time [13], and the range of pore size is restricted which limits the possible applications.

### 2.2. Solid-State Nanopores

Synthetic nanopores, or solid-state nanopores, are fabricated pores drilled in a dielectric inorganic solid membrane. The first materials used were silicon nitride (Si_3_N_4_) and silicon dioxide (SiO_2_) because of the pre-existing expertise in cleanroom microfabrication [43]. It offers the possibility to fabricate nanopores with a good control over size for high-throughput analyses (hundreds of devices at the same time). In 2001, Li et al. reported the first nanopore with a diameter of 1.8 nm fabricated in Si_3_N_4_ thanks to the Focused Ion Beam (FIB) technique [4]. The nanopore was then used to detect single events of DNA going through the nanopore. Another important advance was made when Storm et al. proposed to use a transmission electron microscope (TEM) to fabricate a nanopore in a SiO_2_ membrane [44]. The advantage of TEM drilling is the immediate visual feedback over fabrication. Other techniques of fabrication include scanning transmission electron microscope (STEM) [45], electrochemical removing of atoms in molybdenum disulfide (MoS_2_) [46] or dielectric breakdown [47,48]. The materials used for the dielectric membrane are various, apart from Si_3_N_4_ and SiO_2_, nanopores have been fabricated in thin materials for a higher sensitivity [23] such as graphene [49,50,51,52] or hafnium dioxide (HfO_2_) [53].

Working on fabricated solid-state nanopores has certain advantages over their biological counterparts [54]. They present tunable geometrical designs, a better mechanical stability over a wide range of salt and pH experimental conditions and a possible control over surface chemistry. However, the fabrication process is often long and fastidious. Another drawback is a possible non-specific adsorption onto the surface, leading to pore clogging [19,55,56]. Those problems can be overcome thanks to surface functionalization or original combinations of biological nanopores with solid-state nanopores.

### 2.3. Hybrid or Biomimetic Nanopores

**Figure 3:**
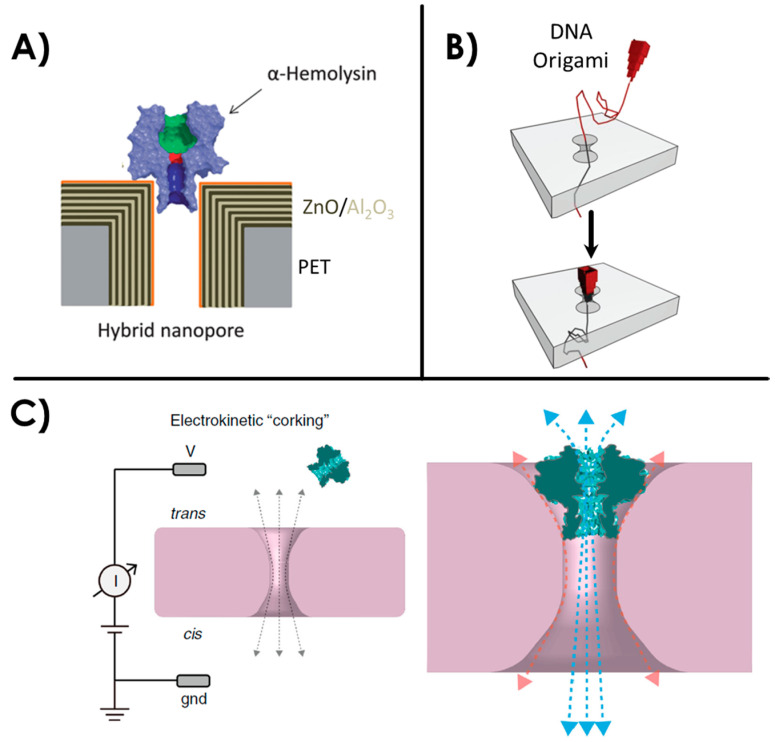
Examples of hybrid nanopores. (**A**) A hybrid nanopore consisting in a α-hemolysin biological pore inserted in a solid-state nanopore, adapted from [58]. (**B**) A DNA origami nanopore. Reprinted adapted with permission from [65]. Copyright 2012 American Chemical Society. (**C**) Virus portal protein inserted in a solid-state nanopore from reference [62].

Biological nanopores such as α-HL possess a precise structure and the potential for site-specific chemical modifications and genetic engineering. It is generally embedded in a lipid bilayer which can be delicate to form and manipulate. On the other hand, solid-state nanopores present a good robustness over time but the reproducibility between each nanopore is more uncertain. The concept of hybrid nanopores combines the advantages of both types. Hall et al. [57] first reported a hybrid nanopore in 2010 with the insertion of an α-HL biological pore in a solid-state nanopore using a DNA guiding tail. The integration of α-HL into solid-state nanopores has since then been performed in other studies [58,59]. To follow in those footsteps, other studies have shown different strategies for hybrid nanopores, such as the grafting of FG-nucleoporins into solid-state membranes to mimic nuclear pore complexes [60,61]. Another example is the insertion of a viral protein portal into a solid-state nanopore membrane [62]. Challenges regarding such hybrid nanopores remain in the control of the protein insertion into the membrane, and the control of possible peripheral leakages around the biological pore. Another interesting approach toward hybrid nanopores is the increasingly popular use of DNA origamis. They offer a good control over shape, size and other functional options [18,63]. DNA origami shaped as a nanopore docked onto solid-state nanopore has already been demonstrated in several studies [20,64,65,66,67,68,69,70] and single translocations of molecules such as proteins and DNA have been performed. Examples of aforementioned hybrid nanopores are illustrated in Figure 3.

After the fabrication of solid-state nanopores on the beginning of the 2000s, interest has grown over their inner surface functionalization [71]. Challenges and objectives met for the functionalization of nanopores can be the same encountered for the functionalization of biochips [72], and many functionalization techniques are inspired from the biosensor’s field. The objectives for nanopore functionalization range from antifouling and anti-clogging properties [73,74,75,76,77] (reduction of non-specific interactions at the pore surface), to the addition of original functionality or offering biomimetic properties [78]. Some recent reviews describe all the different techniques and purposes of surface functionalization in nanopores [24,78,79].

A first technique consists in the controlled deposition of a coating material with a gas phase. It is inspired from microfabrication technologies: atomic layer deposition (ALD) [80] and chemical vapor deposition (CVD) [81]. Such techniques are employed to monitor size and shape of the nanopore [82,83,84], or modify surface properties such as charge or hydrophobicity [82,83]. A good advantage of such a coating is that it can increase the stability of the membrane over time by preventing a slow etching by the electrolyte [85]. In particular, some studies have shown that the gas phase deposition of HfO_2_ has inhibited Si_3_N_4_ dissolution [86].

Another technique consists in the use of surfactants adsorption or physisorption of chemical reagents on the surface. Surfactant can be used on the surface to reduce non-specific interactions of the biomolecule with the pore walls [75,87]. Physisorption can be used as a straightforward technique to coat a nanopore surface. A popular coating using this method is poly-L-lysine (PLL), a positively charged synthetic amino acid chain [88,89]. It also provides the possibility to engineer specific interactions with various proteins [90,91]. Layer-by-layer (LBL) self-assembly is another approach to nanopore functionalization. It consists in the formation of multilayer structure alternating between polyanionic and polycationic layers. The control of the deposition allows a fine-tuning of pore diameter [92], modify the surface physical characteristics [93,94,95] or even combine it with PLL in order to detect specific proteins [95,96]. Another commonly used technique for surface functionalization applied on nanopores is the use of self-assembled monolayers (SAMs). They are composed of molecules bearing a functional group, a thiol for example, which reacts and provokes the grafting on the membrane surface, a gold layer surface for thiols. They are used in nanopore sensing to graft various ligands from DNA, aptamers or proteins and for various applications such as the detection of specific analytes [60,97,98,99] or the addition of a functionality such as pore gating [100,101,102]. Lipid coating of nanopores is an increasingly popular technique due to the biomimetic features it provides [73,103]. It can prevent from non-specific adsorption of analytes to the pore walls, but can also be used to decorate the membrane surface with lipid anchored ligands or receptors such as DNA, aptamers or proteins [79] that are specific to target proteins. Lipid coating allows a reduction of protein’s translocation speed and thus enhance the sensitivity of the sensor [9,73,76].

The most useful surface functionalization technique for SiO_2_ or Si_3_N_4_ membranes is silanization. It involves the reaction of covalent binding between organosilanes molecules with the hydroxyl groups on a surface [104,105], which are created after plasma treatment on SiO_2_ or the thin oxidation layer on Si_3_N_4_ in a solid-state nanopore. A reactive functional group (amine, carboxylic group, epoxide…) in the silane molecule allows the reaction with specific probes. In the nanopore field, silanization has been used to functionalize the surface of nanopores with various biomolecules [79] such as DNA (including aptamers) [106,107,108,109], nucleoporins [61], cystein amino acid [110], peptides [111,112], or pH or temperature responsive polymer brushes [113] and various chemical components [71,114,115,116,117]. In most surface functionalization, the whole surface of the nanopore membrane is covered inducing a loss in recognition specificity inside the pore for sensing applications. Interestingly, the ContactLess ElectroFunctionalization (CLEF) technique allows functionalizing with a large range of ligands (DNA, antibodies) only the inner part of the nanopore [118,119,120,121] which is a good strategy for enhancing the detection of protein with solid-state nanopore [122].

### 2.4. Nanopipettes or Glass Nanocapillaries

In the early 2000, nanopores drilled in synthetic membranes constituted the vast majority of the literature in this domain. However, expensive and time-consuming cleanroom facilities are generally required for nanopore fabrication. In the last decade, nanopipettes or glass nanocapillaries appeared as a cheaper and faster alternative [123,124,125,126,127,128,129]. The geometry of the pore is slightly different, with a cone shape differentiating the cis and trans side of the pore. The fabrication process is simpler since it does not require cleanroom facilities. Briefly, a glass pipette is heated and stretched using a laser micropipette puller. Depending on the parameters, different diameters can be obtained down to tens of nanometers [125]. Silane chemistry has also been performed on glass nanopipettes for electrostatic-gated transport [130]. PLL, which is positively charged, has also been used to adsorb the negatively charged glass surface [89]. Moreover, the use of nanopipettes to detect single molecules in combination with other techniques has been developed. For example, Bulushev et al. have combined nanocapillaries with optical tweezers to discriminate DNA-protein complexes [131]. The optical tweezers served as a handle to have control over the biomolecule translocation speed and allowed precise localization of protein binding sites. Another team has combined nanocapillaries with Surface-Enhanced Raman Scattering for intracellular chemical sensing [132]. Thanks to their ease of fabrication and manipulation in comparison with nanopores, glass nanopipettes are increasingly used to probe the chemical and biological phenomenon down to the nanometer scale.

## 3. Nanopores and Aptamers: A Winning Combination

### 3.1. Aptamers: Molecular Swiss Army Knife

Aptamers are short single stranded oligonucleotides either DNA or RNA specifically selected towards a target. The systematic evolution of ligands by exponential enrichment (SELEX) method [133,134] is an in vitro selection based on a random library of 10^15^ different sequences of oligonucleotides. Generally, the random sequence of 40 nucleotides is flanked by two common primer sequences. The sequences in the library are exposed to the target molecules. Those not binding with the target are removed, whereas the bound sequences are eluted and amplified by Polymer chain reaction (PCR) thanks to the primers. The amplified sequences are then used as the new library for subsequent rounds of selection. Due to an increase of the stringency of the elution conditions, the sequences with the strongest affinity towards the target are selected. After several tens of rounds, the sequencing of the selected library is performed to obtain the best binding aptamers. The binding affinity and the selectivity is principally due to the large variety of initial sequences. Furthermore, secondary structures of the aptamer like hairpins [135,136] and/or G-quadruplex [137,138,139,140,141] are often implied in the binding pocket. The kind of targets for which aptamers have been selected ranges from small ions, organic molecules like pesticides or hormones, peptides and proteins up to cancer cells or bacteria. Depending on the size of the target, the folding of the aptamer may be induced by the recognition with the target. The triggering of the conformation by the target confers to aptamers interesting properties beyond the simple affinity recognition explaining their use in various applications as a simple molecular Swiss Army knife. Moreover, aptamers are easy to produce by automated DNA synthesis and can be functionalized by a wide range of reactive functions such as amine or thiol moieties for further grafting on a surface.

Nanopore sensing has benefited from this trend. Aptamers have been used in numerous studies for the specific detection of target molecules and proteins. To do so, different strategical approaches have been developed. They can be categorized in two broad sections: the single-molecule sensing of the free aptamer complexed with its target in solution or the functionalization of nanopores with specific aptamers. Furthermore, the triggering conformational change of aptamers has also been put forward to provide functions to the nanopores.

### 3.2. Aptamer Structure and Aptamer-Target Studies Using Naked Nanopores

Aptamer’s specificity is based on the 3D spatial conformation of the DNA sequence for the recognition of targets. Since solid-state nanopores support a large range of pH and ionic strength experimental conditions, it is straightforward to adapt the protocol to allow for an optimal folding of the aptamer in its 3D conformation. Usually, the pH and ionic strength conditions for the aptamer selection are close to physiological conditions. Thus, they are also compatible with biological or biomimetic nanopores.

**Figure 4:**
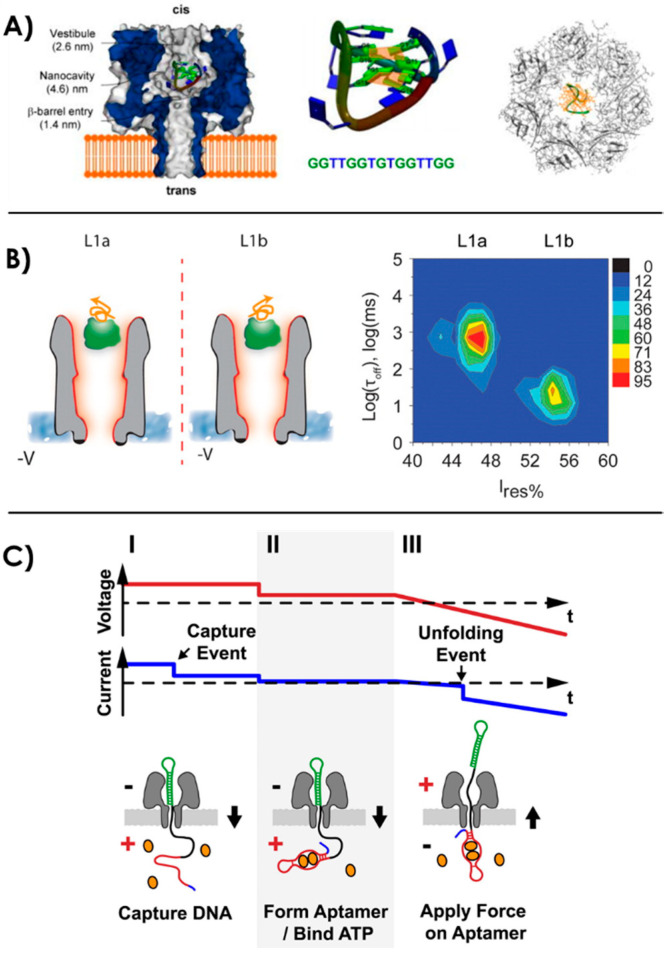
(**A**) Capture of the folded G-quadruplex aptamer in the α-hemolysin nanopore cavity. When linearized, the aptamer is able to go through the narrowest region of the pore and translocates. Reprinted adapted with permission from [139]. Copyright 2008 American Chemical Society. (**B**) Aptamer-ligand conformation study of thrombin-binding aptamer and two possible isomeric configurations demonstrated with a ClyA nanopore. Reprinted adapted with permission from [140]. Copyright 2014 American Chemical Society. (**C**) Nanopore force spectroscopy of an ATP-aptamer complex [141].

Several teams have worked on the fundamental understanding of this conformation and the parameters involved for its stability. In 2007, Thomson et al. assessed the structure of different aptamers in the vestibule of an α-HL nanopore [142,143]. They worked on “Y-shaped” aptamers (TATA sequences for the specific binding of TATA binding proteins) and DNA hairpins. They proposed a statistical analysis method of the current blockage occurring while the aptamer interacts with another molecule, with the channel only, or from undergoing conformational changes [143]. G-quadruplex is a secondary DNA structure formed by interactions between at least four guanines G around a cation. They are notably found in thrombin binding aptamers and are an essential component for the recognition of the target. α-HL nanopores have been used as a tool for the structural studies of G-quadruplex, its kinetics of folding and unfolding and the effects of cation selectivity over the stability of the quadruplex [139,144,145,146,147] (Figure 4A). Shim et al. have demonstrated with the capture and linearization of a thrombin binding aptamer in a nanopore that the structure is more stable in presence of K^+^ ions than other monovalent cations [147].

In 2014 Mahmood et al. proposed a molecular dynamics simulation of a thrombin aptamer in a 6 nm diameter silicon-nitride nanopore [148]. They showed that the 3D structure of the aptamer is more stable under low voltage. Moreover, they worked on the interaction of the aptamer with its thrombin ligand into the nanopore when the aptamer is free or grafted on the nanopore’s wall. They observed with the simulation that the binding affinity is impacted by the applied voltage and that the thrombin translocation time in the nanopore is greater when the aptamer is grafted on the walls. More and more experimental studies assess the interactions between an aptamer and its specific ligands in a nanopore. The thrombin-aptamer association rates have been discussed in several studies [149,150]. Moreover, conformational heterogeneity of the thrombin-aptamer complex has been demonstrated with a ClyA biological nanopore [140]. They showed with the current blockade amplitudes and dwell-times that the complex had two different isomeric conformations, which originate from the aptamer being able to bind two different areas of the thrombin protein (Figure 4B). This proves the opportunity to probe conformational heterogeneity at a single-molecular level of protein-aptamer interactions with a nanopore. Other interactions of proteins with their specific aptamers have been investigated with nanopores, such as nucleocapsid protein 7 [151], a protein biomarker of the HIV-1 virus, or TATA binding protein and HIV DNA integrase [142].

Several other biomolecular interactions with their aptamer have been studied with nanopore technology. ATP interactions with its binding aptamer and the conformation changes with competitive molecules have been assessed [152]. In 2013, Arnaut et al. have used a nanopore as a force spectroscopy device for probing the binding of ATP binding aptamer to its target [141]. They used a backward translocation technique (Figure 4C). They pulled the aptamer-ligand system by one strand captured in the nanopore, the strand can only fully translocate when the complex has been disrupted by the pulling force. Then, they could determinate the dissociation constant K_d_ ≈ 0.1 mM and the voltage dependence of unfolding rates. The higher the ligand concentration, the higher the voltage needed for unfolding of the aptamer-ATP complex, which was called the “critical unzipping voltages of the complexes”. A limit of this force spectroscopy was drawn to attention in this study while doing the same test with the stable thrombin G-quadruplex aptamer. They showed that aptamers with a strong secondary structure could not be tested by this pulling technique because of their highly stable nature.

The differentiation between the interaction of an aptamer and two photoisomeric forms of a molecule have also been demonstrated [153]. Spiropyran and merocyanine are isomers of the same molecule respectively under visible light and ultraviolet light. The spiropyran specific aptamer generates two specific current signatures when going through a nanopore when it is bound to spiropyran or free. It does not interact with the merocyanine form of the molecule. When inserting the aptamer and the spiropyran molecule under visible light, typical current trace of aptamer-ligand complex were observed. Under ultraviolet light, the aptamer dissociated from its target. Finally, the structure and stability of aptamers complexed with chemical compounds targets such as lead [154,155,156,157,158] and mercury [155] ions have also been assessed.

Single-molecule studies with nanopores represent a great tool for the structural study of aptamers when captured in the vestibule of biological nanopores, for example. New strategies for studying the stability of aptamers and aptamer-ligand complexes, their association and dissociation constants as well as their structures under various conditions are being developed, such as the force microscopy reverse pulling technique presented by Arnaut et al. [141]. We can probably expect that nanopore popularity amongst scientists will probably increase for the next years improving the fundamental studies in the field of aptamers.

### 3.3. Aptamers as Carrier Probes for Nanopore Sensing

A strategy for the detection of specific targets consists in the single-event detection of aptamer-ligand complexes going through the nanopore. The aptamer and its target are inserted into the solution and freely associate before going through the nanopore. We can categorize this technique in two approaches: free aptamers in solution or the use of aptamer-functionalized nanoparticles.

#### 3.3.1. Aptamers as Carriers for Nanopore Sensing of Their Target

This category appears as the most straightforward approach of using nanopores and aptamers for the specific detection of targets. Free aptamers in solution have been used for the specific nanopore detection of proteins such as vascular endothelial growth factor (VEGF) [159], thrombin [159] or viruses protein biomarkers [160]. Nucleocapsid protein 7 (NCp7) is a protein biomarker of the human immunodeficiency virus (HIV-1). In a study, NCp7 protein was specifically detected with three different aptamers variants (high, medium and no affinities with the target) [160]. They analyzed single-events of current blockades when the protein-aptamer complex went through the Si_3_N_4_ nanopore and assessed the effect of nanopore dimension (<6 nm or 7–15 nm diameters in a 40 nm thick membrane). As a result, they showed that the detection sensitivity is optimal when the nanopore’s diameter is comparable to the target’s size, hence the cross-sectional size of the NCp7-aptamer complex.

**Figure 5:**
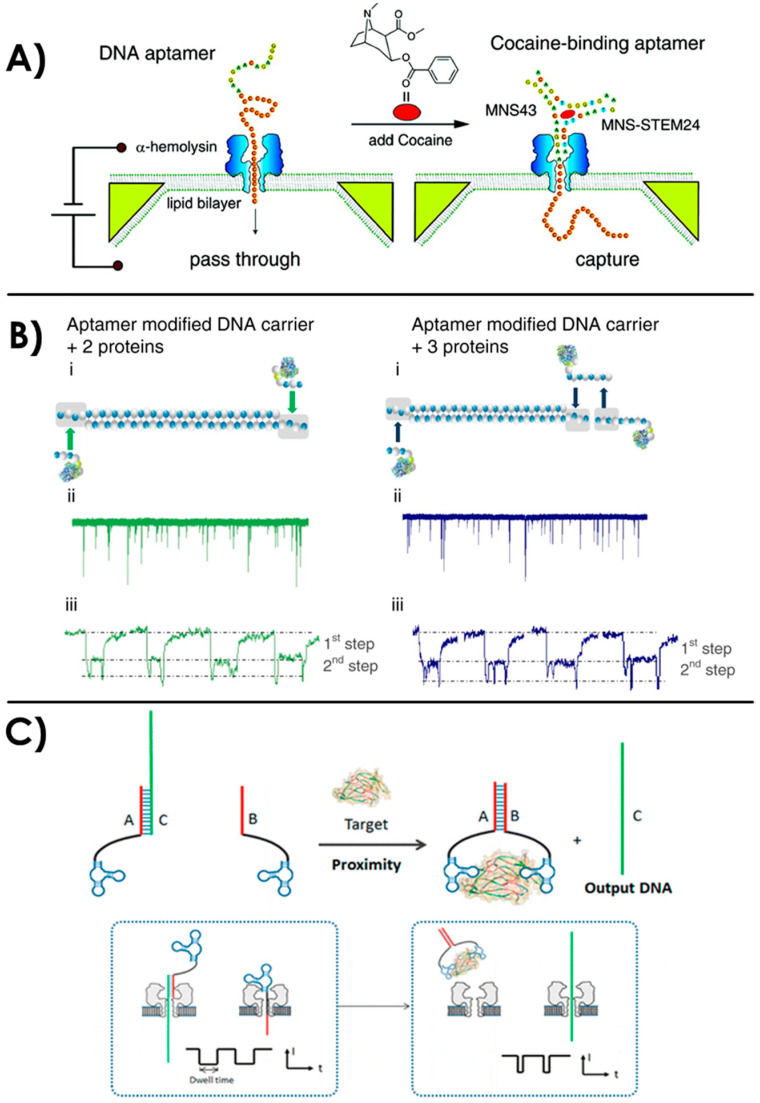
(**A**) Nanopore detection of cocaine with aptamers in solution. Reprinted adapted with permission from [156]. Copyright 2011 American Chemical Society. (**B**) “DNA bar-coding” of a DNA carrier with several aptamers for the specific detection of proteins thanks to the analysis of intra-events [157]. (**C**) Detection of a relatively big target with the release of an intermediate DNA sequence during the aptamer recognition of the target. Reprinted adapted with permission from [158]. Copyright 2015 American Chemical Society.

Other research works involving a nanopore detection of a target with an aptamer in solution concerns small molecules such as ions [154,161], ATP [162], or sensitive compounds such as cocaine [156,159,163,164] or pesticides [165,166,167]. In 2011, Kawano et al. presented an embedded device for a rapid aptamer-based detection of 1 µM cocaine in solution with an α-HL nanopore [156,163]. With the study of current blockades through the nanopore, they observed the difference between the cocaine complexed with the folded aptamer, too large to go through the pore and thus captured, and the free unfolded aptamer when cocaine is not present (Figure 5A). The size of the nanopore compared to the analyte is a key parameter in this study. In the absence of cocaine, the aptamer stays in a linear single stranded DNA conformation and goes through the 1.5 nm constriction of the biological nanopore. They performed the detection of cocaine within a minute on multiple nanopores at the same time, showing that this device could be used for massive and parallel drug detection. A similar approach has been performed by Rauf et al. with an aptamer hybridized with a short complementary DNA [164]. When the aptamer binds to its target, the complementary strand is released and generates a specific current output when going through the nanopore. They could quantify cocaine with a concentration range from 50 nM to 100 μM and proved the selectivity of their device with control molecules (ATP, adenosine diphosphate ADP, dopamine, and theophylline). Moreover, they performed the detection of 50 nM cocaine in human serum and saliva samples, which ensures a great potential practical application of this technique.

Another potential application of the combination of nanopore detection with aptamers is the detection of pesticides in environmental samples. In 2015, Nobukawa et al. presented a strategy for the detection of the pesticide vapor omethoate with an α-hemolysin nanopore and a specific aptamer [165]. They used the same strategy as the previously described cocaine sensor. When the aptamer in solution is bound to its target, it is too large to go through the nanopore and clogs the entry, thus generating a specific current signature. Later, the same group has added a hydrogel to absorb and detect the organic volatile compounds from the vapor phase [167]. They exposed the hydrogel to vaporized omethoate at a concentration of 100 ppb (part per billion) for 10 min, resulting in an absorption of 600 nM vaporized omethoate and its detection with the aptamer and the nanopore. The same device could detect a concentration down to 4.8 nM of omethoate in solution. Then, they notably improved the permeation of the vapor compound into the analyzed solution while keeping the same detection strategy of the pesticide [166].

The size ratio between the target bound to the aptamer and the nanopore diameter is a key parameter to consider for the nanopore detection of aptamer-ligand complexes. In the past few years, several new strategies emerged to cast off this constraint, such as the “DNA bar-coding” technique [168]. It consists in the labeling of long DNA strands with spatially controlled markers, giving a specific current signal with recognizable intra-events. The combination of this technique with aptamers has allowed the detection of ATP [169,170] and proteins [157,170]. In 2017, a double-stranded DNA scaffold assembly with an aptamer protrusion has been used for the specific detection of ATP [169]. Therefore, when going through the 3 nm diameter silicon nitride nanopore, the DNA strands generate a unique signature when ATP is bound to the aptamer protrusion. The same year, another study has shown the possibility to use an aptamer-modified DNA carrier for the “bar-code” detection of different proteins [157]. λ-DNA, a long double-stranded DNA with a standardized sequence have been used as the DNA carrier for its rigidity and reproducible current signature. It has been modified with aptamers onto specific spatial locations. When the target thrombin protein bound to the aptamers, sub-peaks in the current blockade with up to three targets on the same carrier could be observed (Figure 5B). A thrombin concentration down to 1.6 nM could be detected. Moreover, this technique has been extended to multiple target proteins in the same solution on the DNA carrier. Different aptamers on the same carrier were combined (thrombin aptamer and an enzyme acetylcholinesterase aptamer). One must notice that they needed to adapt the size of the nanopore to the desired target. Finally, they performed this strategy in human serum, demonstrating the flexibility and the efficiency of this bar-coding aptamer detection even in complex sample. Another study has shown the feasibility of using this technique for the simultaneous detection of three different targets on the DNA carrier: ATP, thrombin and lysozyme [170].

Another strategy involving aptamers and an intermediate DNA sequence is emerging as a solution to the size ratio challenge between the nanopore and the analyte. The detection is achieved indirectly by the quantification of the intermediate sequence released when the aptamer and the target form a complex. This represents the great advantage to offer a detection independent from the target’s size and based on the already well-established detection of single-stranded DNA through nanopores [5]. Zhang et al. have demonstrated this strategy with an α-hemolysin nanopore for the detection of platelet-derived growth factor with two B subunits PDGF-BB [158]. The specific aptamer can bind the target on two distinct protein emplacements. An intermediate DNA, called the output DNA was designed to partially hybridize with the aptamer. When the target was added into the solution, the aptamer would bind it and release the output intermediate DNA, which offered a very specific current signature when going through the pore (Figure 5C). With this strategy, they were able to detect PDGF-BB with a limit of detection of 500 fM. They tested the selectivity of the detection with other control proteins (BSA, thrombin, human immunoglobulins G, and glucose oxidase) and further validated the results in 10% diluted human serum. This DNA intermediate strategy has also been used for the detection of a larger target: *Bacillus thuringiensis* spores [171,172]. In this study, the problem of sensing a large target was overcome by the use of intermediate DNA hairpins that binds the spore-specific aptamers. The unique current signature of the released DNA hairpin in an α-HL nanopore allowed the specific detection of the spores. They reported an enhancement of the signal-to-noise ratio thanks to the sample preparation and the analysis of the solution containing only the unbound DNA hairpin intermediates.

The nanopore detection of specific targets with free aptamers beholds great promises for applications in the healthcare domain (detection of drug compound in human samples) or in environmental safety (detection of pesticides). With the inventive bar-coding approach, it is notably possible to sense multiple targets at the same time with a great sensitivity. However, this approach is limited to the detection of small molecules or specific care must be taken into the ratio between the target size and the nanopore diameter. If the desired target is relatively large, another approach has emerged with the sensing of an intermediate DNA sequence released when the aptamer-ligand complex is formed, this allows the sensing technique to be freed from this size limitation.

#### 3.3.2. Aptamer-Functionalized Nanoparticles as Carrier Probes for Nanopore Sensing

Another strategical approach for the detection of specific targets with an aptamer consists in the use of a nanostructure holding the aptamers. The detection via aptamer-coated nanostructures (nanobeads, nanorods) can sometimes require a setup with a relatively larger nanopore (several hundreds of nanometers).

**Figure 6:**
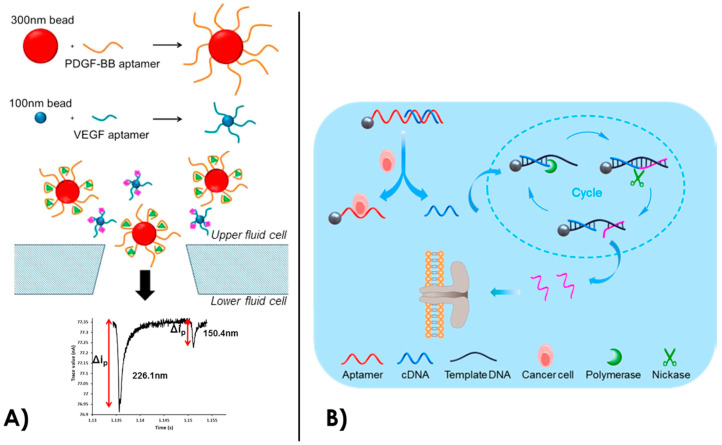
(**A**) Multiplexed Tunable Resistive Pulse Sensing (TRPS) of aptamer coated beads for the detection of PDGF-BB and VEGF on 300 nm and 100 nm beads, respectively [172]. (**B**) Detection of human lymphoma cancer cells by combination of aptamer-coated particles, enzymatic amplification of DNA and biological nanopore detection. Reprinted adapted with permission from [173]. Copyright 2018 American Chemical Society.

In 2012, proteins with aptamer-functionalized particles aggregates were specifically detected with a nanopore [173,174]. 300 nm thick and 1 µm long rods with gold and nickel segments were functionalized with PDGF-BB aptamers in a spatially controlled way. Therefore, they could obtain a precise agglutination pattern between the aptamer-rods after addition of the target PDGF-BB protein. They could detect the target with a concentration down to 10 fM and validated the specificity with BSA as a control protein. Later, they detected 128 nm superparamagnetic beads coated with streptavidin and biotin bound thrombin-specific aptamers [149]. After addition of thrombin in a range of concentration from 0.1 nM to 1 µM, they observed a decrease in the event rates sensed in the nanopore. The specific binding of thrombin on the beads resulted in a shielding effect of the negative charges that drives the beads through the nanopore. They also employed another technique to detect thrombin via the monitoring of aggregates disruption [175]. A mix of superparamagnetic beads of 1 µm coated with thrombin aptamers and 400 nm-beads coated with the complementary aptamers forms aggregates. After addition of thrombin down to sub-picomolar concentrations, the aggregates were disrupted and specific single-event signals were observed with the nanopore. In another study, they used 120 nm diameter streptavidin coated beads functionalized with biotin-bound VEGF aptamer to detect VEGF proteins with a concentration down to 18 pM [96]. In 2015, they developed a strategy for the simultaneous detection of PDGF and VEGF [172]. They grafted VEGF aptamers and PDGF aptamers to 120 nm and 300 nm superparamagnetic beads, respectively. After addition of VEGF and PDGF at the nanomolar scale, the frequency of bead translocation through the nanopore and the current blockade level of each event could be related to each type of protein and their concentration (Figure 6A). This study proved the possibility of simultaneous label-free detection of proteins with aptamer coated nanoparticles.

Other groups have worked on aptamer-functionalized nanoparticles for the detection of biomolecules. In 2017, nanopore sensing of lysozyme was performed using 21 nm diameter quartz nanopipettes and 5 nm aptamer-functionalized gold nanoparticles [176]. They detected lysozyme with a concentration of 250 nM in a solution with background control proteins cytochrome C and trypsin. Alsager et al. have sensed 17β-estradiol hormone by using 217 nm-sized carboxylated polystyrene nanoparticles coated with aptamers [177]. Thanks to the analysis of single-events amplitude, they observed the diameter increase of the nanoparticles after grafting of the aptamer. Then, they also observed the diameter decrease resulting from the conformational change of the aptamer bound to its specific ligand. They reported a detection of 17β-estradiol in the nanomolar range.

Nanopores and aptamer-coated nanoparticles have also been investigated for the detection of polluting agents in water. Microcystin-LR (MC-LR) is a lethal cyanotoxin produced by cyanobacteria in fresh or saline water. He et al. have developed a strategy in 2018 to detect this toxin with two different sizes of gold nanoparticles coated with aptamers and a 20 nm silicon nitride nanopore [178]. They grafted MC-LR specific aptamers on 5 nm gold nanoparticles and the complementary sequence on 20 nm gold nanoparticles. Those two kinds of particles formed aggregates by complementary hybridization of the aptamers. After addition of the target toxin, the aggregates were disrupted and the 5 nm particles with the captured toxin were sensed through the nanopore. They tested this strategy with a concentration range of MC-LR from of 0.1 nM to 20 μM. Additionally, they proved the specificity of their approach with a mixture containing MC-LR, other congener toxins and chlorophyll that abundantly coexists in water. The same year, Mayne et al. have designed aptamer-modified nanoparticles for the detection of mercury (Hg^2+^) and lead (Pb^2+^) ions which are agents of metal pollution in sea-water [155]. With a study of translocation velocities, driven by the aptamer charge density around the 150 and 300 nm particles when bound to their target or not, they could simultaneously detect the two targets in a concentration range between 10 to 200 nM. They also engineered a dual aptamer aiming to detect both Hg^2+^ and Pb^2+^ at the same time.

Recently, there has been an increase of interest toward aptamer-coated nanoparticles and nanopores sensing for biomedical and diagnostic applications. Healey et al. have developed a system for a rapid quantification of prion PrP^C^ proteins [179], which are involved in neurological diseases. After functionalization of 125 nm superparamagnetic beads with PrP^C^ specific aptamers, they specifically detected PrP ^C^ proteins (50 nM concentration) with tunable resistive pulse sensing (TRPS) and the monitoring of particle velocities. They also added 200 nM albumin, fibrinogen or γ-globulin as control proteins to show the specificity of their detection. The entire workflow from cellular extraction to the quantification of prion proteins takes less than an hour and offers a great promise toward the use of nanopore and aptamer coated particles for rapid diagnostics. Another biomedical application concerns the detection of cancer biomarkers. Li et al. modified magnetic nanoparticles with aptamers that target carcinoembryonic antigen (CEA) [180], a cancer biomarker. After addition of the magnetic aptamer-nanoparticles (~5 nm diameter) in a complex human sample containing 1 nM of CEA, the nanoparticles could be magnetically separated for the analysis. Then, a nanopore analysis was performed with a 30 nm diameter quartz nanopipette. By analysis of single events current blockades of particles going through the pore, they could discriminate the presence of CEA on the nanoparticles. Indeed, aptamer-nanoparticles with bound target CEA are three times larger in volume than aptamer-nanoparticles only and could easily be distinguished in the current signal. They tested this strategy in different human serums and compared the results with classical ELISA assays. This technique obtained the same level of performance and could be used for early diagnosis of cancer. In 2020, another study has shown the simultaneous nanopore detection of VEGF, PDGF-BB and thrombin (that are cancer lung biomarkers when overexpressed) with three different nanoparticle-aptamer and complementary DNA constructs [181]. This approach has been further developed in the same group by Xi et al. for an ultrasensitive detection of cancer Ramos cells, which are involved in human lymphoma [173]. They coated 1 µm-diameter beads with aptamers that specifically target Ramos cells. The sequence was hybridized with a short complementary DNA. When the aptamer specifically recognizes the cancer cell, the short complementary strand of DNA is released. Afterward, this sequence is specifically amplified via enzymatic cycling with phi29 DNA polymerase and the collected solution is further detected in an aerolysin biological nanopore. The illustration of their strategy is found on Figure 6B. The output DNA going through the nanopore produced characteristic single events of current blockades and permitted a detection of the Ramos cells with an excellent sensitivity. Down to five Ramos cells could be detected in 100 μL, they also successfully performed the detection into human serum. This approach of sensing an intermediate product released with the target-aptamer binding allows an emancipation from the target size dependence of nanopore sensing. Moreover, this is a good example of selective and precise detection of cancer cells in human serum, which offers a powerful tool for biomedical research and diagnosis.

Aptamer-coated nanostructures have been used for various applications such as water pollution and contaminant detection or advanced biomedical diagnostics. In most of the cases, the ligand-aptamer nanostructure is relatively large (~100 nm) and TRPS can be used with a commercial apparatus called the qNano. The single events of particles going through the pore are analyzed to discriminate the size and the velocity of the nanoparticles according to the presence of the target or not.

Even though monitoring the nanoparticle functionalization can be a technological challenge, using a nanoparticle-coated aptamer instead of a free aptamer in solution could allow an easier nanopore detection thanks to larger objects. Nevertheless, there is still no study that thoroughly compares those two approaches and further examination would be required.

### 3.4. Sensing with Aptamer-Functionalized Nanopores

Over the past years, the grafting of aptamers inside nanopores has been an extensively used approach to detect specific molecules, proteins or dangerous substances such as drugs or toxic molecules. Solid-state nanopores are mainly employed but several cases of genetically or molecularly engineered biological nanopores combined with aptamers have also been reported.

The main physical phenomenon employed for measurements with aptamer-coated nanopores is ionic current rectification (ICR). Nanopores usually exhibit a linear current-voltage (I-V) curve following an ohmic behavior. However, it has been shown that this I-V curve is no more linear when the pore is conical (asymmetric) or has a non-homogeneous fixed charge distribution on the pore walls [182,183,184]. Such ICR phenomenon is used as an indicator for the modification of nanopore’s surface and grafting of charged biomolecules.

**Figure 7:**
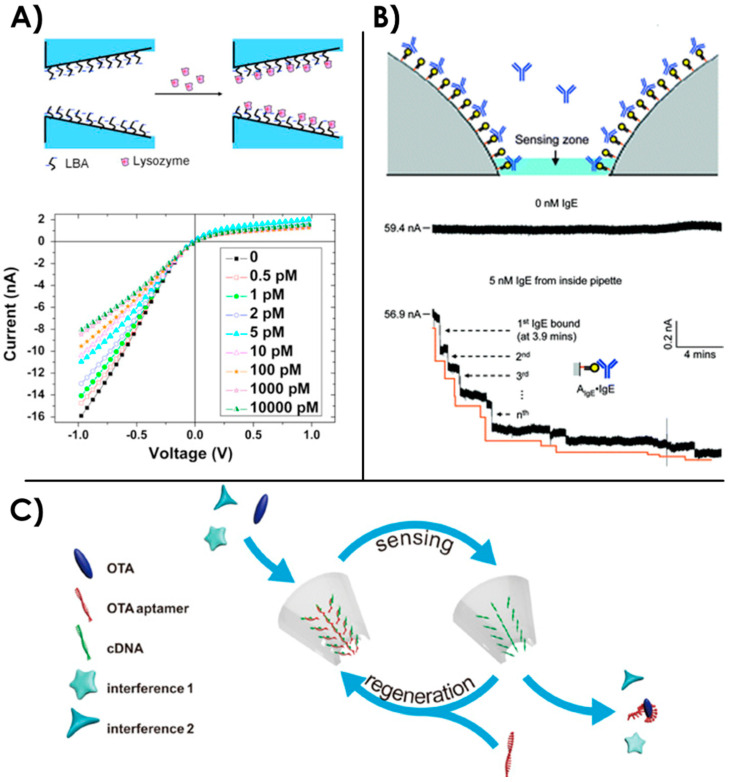
(**A**) Illustration of lysozyme binding aptamer grafting on a 20 nm conical glass nanopores and induced ICR after addition of various concentrations of lysozyme protein [185]. (**B**) Consecutive current decrease steps with immunoglobulin binding on the surface aptamers from reference Reprinted adapted with permission from [108]. Copyright 2009 American Chemical Society. (**C**) Strategy for OTA toxin detection with DNA direct immobilization of aptamers from reference [187].

Specific detection of lysozyme protein with aptamers was performed in a single conical glass 20 nm nanopore thanks to ICR [185]. After the grafting of lysozyme binding aptamers on the inside of the nanopore, ICR was observed due to the negatively charged walls. After the binding of the protein, the global surface charges were partially neutralized and the ICR effects decreased (Figure 7A). Thanks to this, Cai et al. could specifically detect lysozyme amongst other control proteins (BSA, cytochrome c and pepsin) with a limit of detection of 0.5 pM. A similar approach was performed on a 20 nm track-etched nanopore in polyethylene terephthalate (PET) membranes for the specific detection of lysozyme down to 70 µM [186].

Several studies show the interest of aptamer-coated nanopores for the specific detection of thrombin proteins [90,187,188,189]. A 30 nm diameter glass conical nanopore has been functionalized with thrombin binding aptamers and tested with 50 pM of target thrombin in undiluted serum [188]. Successive decreases in the current level has shown the consecutive binding of thrombin proteins on the aptamers. With a probabilistic model, they showed that this device can be efficiently used for quantification of the target with a response time of 10 min. Another group has presented a similar device with a 50 nm quartz conical nanopore coated with thrombin binding aptamers [90]. With a measurement of ICR, thrombin could be detected at a concentration of 270 nM in phosphate buffered saline (PBS) while the control protein bovine serum albumin (BSA) did not affect the current levels. They reported that using undiluted serum would clog their device. Another approach has been performed by Zhao et al. [189] with the use of a 100 nm thick porous anodic alumina membrane with an array of nanopore (~40 nm average diameter). After grafting of thrombin aptamers on its surface, they monitored ICR induced by thrombin binding in 1 mM KCl with different pH conditions. They obtained a limit of detection of 0.22 fM for specific pH conditions. Moreover, they showed that in human serum they could detect thrombin with a concentration down to 0.111 nM.

This could indicate that using nanopore arrays and nanoporous membranes instead of single nanopores could be an alternative to enhance the measurements when using ICR as an indicator for protein detection. However, with arrays instead of single nanopore, the possibility for single-molecule sensitivity is lost. Blundell et al. have used both ICR and single-event measurements in the same study for the aptamer detection of VEGF [96] in a relatively large nanopore (800 nm). In one part of the study, they coated the nanopore with VEGF aptamer and measured ICR to monitor VEGF concentrations down to 5 pM.

Aptamer-coated nanopores are also employed for the detection of proteins such as immunoglobulin [108,190] or the extremely toxic ricin protein [108,191]. Gao et al. have focused their works on the detection of ricin [108] that can be used as a bioterrorism agent. They also used the same kind of device in their study for the detection of immunoglobulin proteins. They functionalized a 56 nm glass nanopore with ricin binding aptamers. After addition of ricin at a concentration of 100 nM, they could monitor the decrease of current when each protein bound to the aptamers. They performed the same experiments with immunoglobulin E specific aptamers (Figure 7B) and could detect the protein with a concentration down to 5 nM. This particular example shows the possibility to use aptamer-coated nanopores as a versatile tool for both diagnostic purposes and environmental detection of dangerous substances.

In this applied field of research, nanopores coated with aptamers has also been used to detect small toxic molecules: cocaine [192,193] and ochratoxin A (OTA) [187], a dangerous product of fungi species that can be found in agricultural products. Wang et al. have performed cocaine detection over a wide range of concentration down to 1 nM [193] in a 30 nm diameter track-etched nanochannel in PET. They used a couple of cocaine specific aptamers that enclosed the target between the two DNA strands. One of the aptamers was immobilized inside the nanochannel, while the second aptamer was inserted simultaneously with cocaine in the solution. They monitored cocaine capture on the surface of the nanochannel thanks to ICR measurements and validated the specificity of their detection with other small control molecules (glucose, atropine, and tropinone). Recently, Zhang et al. have developed a different strategy for the detection of OTA toxins with an aptamer coated nanopore [187]. In a 90 nm diameter glass conical nanopore, they grafted a first DNA sequence that is partially complementary to the OTA binding aptamer. The latter aptamer is hybridized with the pre-immobilized sequence, following the principle of DNA-directed immobilization [194]. After addition of the OTA, the aptamer that partially pairs with the immobilized DNA sequence will bind to OTA and subsequently dissociate from the nanopore surface (see illustration on Figure 7C). Therefore, ICR is generated by the aptamer dissociation that causes a negative charge reduction on the nanopore inner surface. An interesting point for this method is that the ICR measurement is independent from the charge of the target biomolecule, which is an undeniable advantage for this type of detection. Moreover, they showed a simple regeneration of the device by adding a solution containing the OTA binding aptamer for its immobilization on the grafted partially complementary DNA sequence.

Other aptamer coated solid-state nanopores have been used for the detection of adenosine triphosphate ATP [195,196], potassium [196] or TATA box binding protein [197]. Nevertheless, the grafting of aptamers on the surface of a nanopore for the detection of a target is not restricted to solid-state nanopores. Genetic and molecular engineering allows the modification of biological nanopores as well.

**Figure 8:**
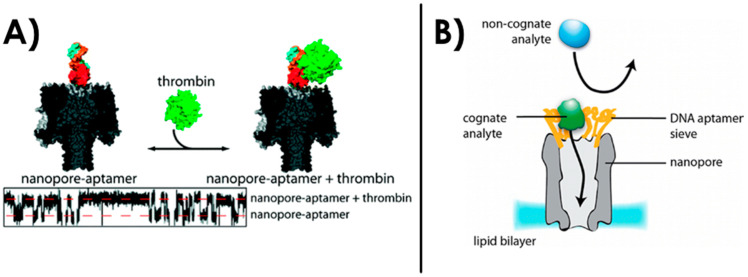
(**A**) α-HL biological nanopore engineered to specifically detect thrombin with a thrombin aptamer at its entrance. Reprinted adapted with permission from [198]. Copyright 2012 American Chemical Society. (**B**) Engineered ClyA nanopore with an aptamer sieve for the specific detection of proteins. Reprinted adapted with permission from [199]. Copyright 2012 American Chemical Society.

In 2012, an α-HL biological nanopore was engineered by Rotem et al. to specifically detect thrombin with an aptamer [198] (Figure 8A). With the analysis of current blockades, they showed that the insertion of thrombin with a concentration down to 20 nM could generate a specific current signature. They also defined specific equilibrium dissociation constants K_d_ that are consistent with the ones obtained using standard approaches. The selectivity of this sensor toward the natively folded thrombin protein was proved using a denatured form and BSA as negative controls.

Later the same year, Soskine et al. have reported an engineered ClyA biological nanopore that exhibited a sieve of aptamers for the specific recognition of proteins (thrombin or lysozyme) [199]. They grafted aptamers to the ClyA monomers, resulting in an assembled nanopore that contains 12 aptamers at its entrance that are ~2 nm apart from each other (Figure 8B). By analysis of the single events of current blockage through the nanopore, the decorated nanopore showed an excellent specificity toward the target analyte even when another protein was introduced. For example, with a thrombin aptamer decorated ClyA nanopore, a control protein was added in large excess concentration compared to the specific human thrombin target (2.2 nM) but they managed to record twice as much thrombin specific events compared to the control protein. With this nanopore, they succeeded in mimicking the nuclear pore complex selectivity of translocated molecules, which is one of the goals of engineering nanopores for biomimetic applications. Another study using aptamer-functionalized nanopipettes in combination with surface-enhanced Raman scattering showed localization of cancer biomarkers inside single cells [200].

As a conclusion, specific detections of targets have been performed on aptamer-functionalized nanopores thanks to ICR measurements and successive current decreases when each target binds the inside of the nanopore. ICR is a great indicator but is generally dependent on the target’s charge. An interesting variation of this sensing strategy was presented by Zhang et al. with DNA-directed immobilization of the aptamer for the detection of OTA toxin [187]. With the release of the aptamer-OTA complex from the nanopore walls, the ICR measurement was independent from the target’s charge and the device could be easily regenerated for further experiments. There are some examples of aptamers grafted on biological nanopores. In this case, the monitoring of single events of biomolecules going through the nanopore is then used for the specific recognition of a target.

**Figure 9:**
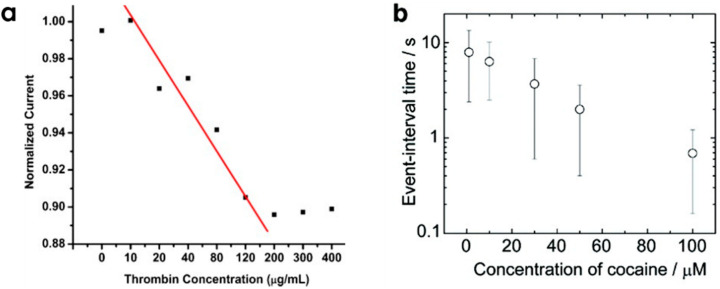
(**a**) Calibration curve of a protein (thrombin) quantification based on the normalized current from nanopipette sensing. Reprinted adapted with permission from [90]. (**b**) Calibration curve of a small molecule (cocaine) from the blocking time by the aptamer bound cocaine. Reprinted adapted with permission from [156]. Copyright 2011 American Chemical Society.

The various nanopore strategies developed in those two sections for the sensing of targets should be compared with the conventional biosensors using aptamers also called aptasensors. In aptasensors, the aptamers are generally grafted onto a surface and the recognition events are analyzed thanks to transducing techniques (optical, electrochemical or mechanical). The main advantage of the nanopore strategies is the possibility to detect single events. On this respect, nanopores are stochastic detection sensors where the statistic of events would finally give information on the amount of targets in the solution (Figure 9) whereas standard aptasensors are based on averaged results based on multiple recognition events. Concerning the limit of detection of targets, the possibility of detecting single events is a great asset to nanopore sensing. However, size reduction of conventional aptasensors recently converged to the possibility of single event recognition. Concerning the calibration curves for target quantification, all strategies are mainly constrained by the size of the target and its affinity with the aptamer.

### 3.5. Gating with Aptamer-Functionalized Nanopores

**Figure 10:**
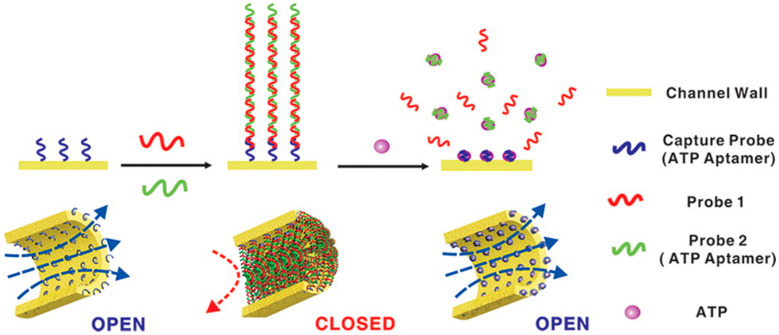
Highly-efficient gating with two ATP aptamers forming a super-sandwich structure in a nanochannel. Reprinted adapted with permission from [201]. Copyright 2012 American Chemical Society.

In 2012, Jiang et al. presented an array of nanochannels of ~60 nm diameter in alumina membranes with an aptamer structure allowing a closed and open state [201]. The inner surface of the nanochannel had been grafted with a first capture ATP aptamer probe. Then, two other sequences could hybridize on this probe, forming an aptamer super-sandwich structure that obstructed the channel as a perfect electric seal (∼GΩ). After addition of ATP in the system, the aptamer assembly into the channel was disrupted by the ATP-aptamer recognition and the ionic current was established. The channel was then in its open state (Figure 10). With those open and close states, they were able to perform logical operations with eight parallel structures, showing the opportunities for complex nanofluidic architectures and manipulations. Later, the same group studied the effects of asymmetric exposure of this system to ATP both experimentally and theoretically [183]. They probed different ICR profiles between the open or closed stated when the channel was partially closed by the aptamers.

Recently, Acar et al. reproduced potassium selectivity inside a solid-state nanopore using crown ether and single-stranded DNA grafting [202]. The device was tested using nanopores of different diameters, at different salt concentrations and under a 1V voltage range. Current measurements with a mixture of KCl and NaCl at different ratios showed that the functionalized nanopore acted as a gate towards sodium ions, selectively letting potassium ions through.

Other aptamer-coated channels have been reported to respond to small molecules such as cocaine [192] or adenosine [203] in a reversible manner. In the latter case, the study was inspired by natural adenosine receptors found on the cell’s membrane and responsible for cellular signaling pathways. Polymer membranes with ion-track etched nanopores (20 nm) were coated with adenosine aptamers [203] that filled the inside of the channel. After addition of adenosine, the aptamers folded in a compact way, allowing the flow of the ionic current. They could reverse the process and close the channel again by addition of adenosine deaminase, an enzyme which converts adenosine into inosine molecules that are no more recognized by the aptamers.

## 4. Conclusions and Perspectives

**Figure 11:**
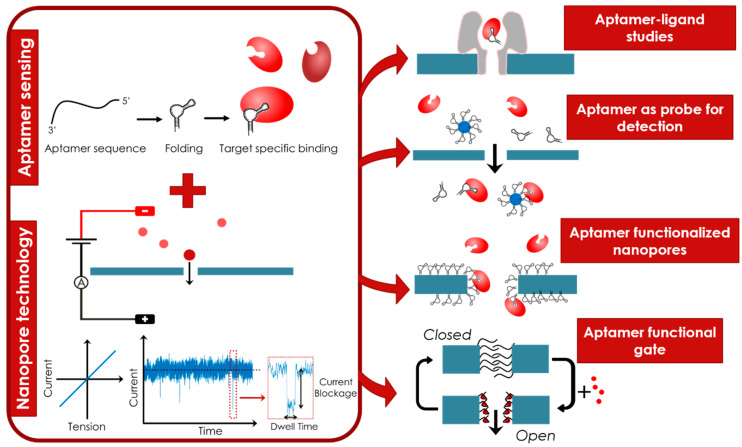
Schematic summary of the review. The winning combination of aptamer sensing with nanopore technology opens the door to fundamental studies of aptamer-ligand structures, target detection through recognition by aptamers in solution or functionalized in the nanopore aperture and finally to functional nanopores due to the conformational switching of aptamers.

Either free in solution or grafted onto the nanopore, aptamers are generally used as recognition elements to improve the sensibility and/or the selectivity of nanopores to detect a target (Figure 11). Thanks to the versatility of the aptamers, the range of accessible targets is large, from simple ions, pesticides, hormones or proteins to name a few. This wide range of targets allows for applications in various domains like health (diagnostics or drug discovery), environment (water pollution) and security (drug detection). The ease of production and molecular modifications along with the low cost of aptamer chemical synthesis and the huge stability of aptamers are good assets for the industrialization of functionalized nanopore devices. The combination of aptamer sensing with nanopore technology is therefore a winning association promised to numerous developments.

Besides, nanopore or nanochannel gating has raised interest as nanofluidic valves or biomimetic selective transport tools. Mimicking the selectivity of gating functions from the biological ion channels can allow a controllable release of molecules into a fabricated system. A lot of different approaches already exist for stimuli-responsive gating of nanopores [78]. Aptamer-coated nanochannels represent a new strategy for the precise control of gating systems responding to biomolecule stimuli.

Synthetic ions channels and stimuli responsive gates are one of the first steps toward nanoscale sensors and actuators. The aptamer gates and setting of nanometric logic operations represent good candidates for potential advanced structures called molecular robots [204] or nanoscale power generator [205]. Those molecular robots could represent a promising tool for environmental monitoring or healthcare applications such as in vivo diagnosis or drug delivery. The high selectivity brought by aptamers could also serve in separation membranes [206,207] for water desalination [208,209,210] or wastewater treatment [211]. Those are only few directions where biomimetic nanoporous membranes could be useful in the future [212].
